# The effect of insulin analogs in people with type 1 diabetes at increased risk of severe hypoglycemia

**DOI:** 10.3389/fphar.2023.1301931

**Published:** 2023-11-27

**Authors:** Sofie Broeng-Mikkelgaard, Julie Maria Bøggild Brøsen, Peter Lommer Kristensen, Birger Thorsteinsson, Ulrik Pedersen-Bjergaard

**Affiliations:** ^1^ Department of Endocrinology and Nephrology, Copenhagen University Hospital, Hillerød, Denmark; ^2^ Department of Clinical Medicine, University of Copenhagen, Copenhagen, Denmark

**Keywords:** type 1 diabetes, insulin analogs, hypoglycemia, randomized clinical trials, insulin lispro, insulin glargine, insulin detemir, insulin degludec

## Abstract

Type 1 diabetes is characterized by insulin deficiency, and treatment is to supply insulin mimicking the physiological endogenous insulin secretion. Since its discovery, insulin therapy has evolved, and since the 1990s, an increasing number of insulin analogs with various pharmacokinetic and pharmacodynamic profiles have become available. Despite the improvement of insulin therapy, hypoglycemia remains the main side effect and is a daily concern for many people with diabetes and their families. A proportion of people with type 1 diabetes are at increased risk of hypoglycemia and experience recurring episodes. When designing insulin trials, this group of people is most often excluded in order to reduce the risk of adverse study outcomes, even though it may be the group that may benefit the most from treatment with new insulins. The results of the phase III trials, therefore, underestimate the clinical impact and pharmacoeconomic effect of the implementation of new insulins in the broader type 1 diabetes population. This paper reviews the four insulin trials that include people at increased risk of hypoglycemia. In general, the studies confirm the results from phase III trials in terms of similar reduction and maintenance of HbA1c, as well as relative rate reductions of hypoglycemia. However, the absolute treatment differences in the reduction of hypoglycemia are even greater in the trials, including people at high risk of hypoglycemia. This emphasizes the importance of including people at high risk of hypoglycemia to assess the full clinical and pharmacoeconomic benefit of new insulins.

## 1 Introduction

The cornerstone in the treatment of type 1 diabetes is to supply insulin, mimicking the physiologic endogenous insulin secretion as closely as possible. In people with normal beta cell function, insulin secretion is a complex mechanism that can be deconvoluted into two components: basal insulin secretion, which maintains metabolism and normal glucose levels during fasting (such as during overnight fast and between meals), and prandial insulin secretion in response to carbohydrate intake (Hirsch et al., 2020). The standard insulin therapy regimen in people with type 1 diabetes is either basal-bolus therapy or the use of insulin pumps. Basal bolus therapy is based on long-acting insulin to replace the basal insulin secretion and rapid-acting insulin to replace prandial insulin secretion—also known as multiple daily injections (MDI) treatment. Continuous subcutaneous insulin infusion (CSII) via an insulin pump deliver rapid-acting insulin with a continuous basal rate—either based on pre-programmed basal rates or algorithm-directed insulin delivery based on glucose sensor values (automated insulin delivery) and user-initiated prandial and correctional boluses (Kesavadev et al., 2020).

The main side effect of insulin therapy is hypoglycemia, which occurs when the insulin action exceeds the physiological need (Cryer, 2014). The severity of hypoglycemia can be classified into non-severe self-treated episodes, which can range from asymptomatic to mild symptomatic episodes, and severe episodes where external assistance is needed to restore glucose levels (Graveling and Frier, 2010; Amiel, 2021). The risk of hypoglycemia is a major concern for people with insulin-treated diabetes and their relatives. The fear of hypoglycemia can become a barrier to achieving good glycemic control, and this may, in turn, increase the risk of diabetic complications (Cryer, 2008; Cryer, 2014). Furthermore, severe hypoglycemia is linked to an increased risk of cardiovascular disease and mortality (Amiel et al., 2019).

Impaired awareness of hypoglycemia is the reduced ability to perceive and detect symptoms of hypoglycemia (Graveling and Frier, 2010). Recurrent hypoglycemia can result in impaired awareness of hypoglycemia by lowering the glucose levels for which both symptoms of hypoglycemia and counter-regulatory responses are evoked, which can lead to a vicious cycle of recurrent hypoglycemia and an increased risk of both asymptomatic and severe hypoglycemia (Cryer, 1993). It is estimated that 20%–40% of all people with type 1 diabetes have impaired awareness of hypoglycemia (Geddes et al., 2008; Lin et al., 2020). People with insulin-deficient diabetes who have impaired awareness of hypoglycemia have a more than six-fold higher risk of severe hypoglycemia (Cryer et al., 2006) and a 2-4-fold higher frequency of asymptomatic hypoglycemia than people with intact hypoglycemia awareness (Graveling and Frier, 2010). In an unselected population, up to 20% of all people with type 1 diabetes have recurrent severe hypoglycemia (Kristensen et al., 2012a). These people at increased risk of hypoglycemia need near-physiologic insulin replacement to reduce exposure to hypoglycemia.

Since the first successful injection of purified animal-extracted insulin, insulin therapy has undergone a massive development (Table 1) (Hirsch et al., 2020; Bolli et al., 2022). Today, the therapy is primarily based on synthetic insulin analogs with improved pharmacodynamic properties to fit the requirements of either a stable basal insulin or a rapidly absorbed prandial insulin (ElSayed et al., 2022).

**TABLE 1:** Overview of insulins and their characteristics.

Insulin type	Onset time	Peak effect	Duration of action
Ultra-rapid-acting			
Faster insulin aspart	4 min	1–3 h	3–5 h
Ultra-rapid insulin lispro	2 min	1–2 h	4.5 h
Rapid-acting			
Insulin lispro	10–15 min	1–2 h	3–5 h
Insulin aspart	10–15 min	1–3 h	3–5 h
Insulin glulisine	10–15 min	1–2 h	2–4 h
Short-acting			
Regular human insulin	30 min	2–3 h	6.5 h
Intermediate-acting			
NPH insulin	1–3 h	5–8 h	18 h
Long-acting			
Insulin Glargine U100	1.5 h	None	24 h
Insulin Glargine U300	6 h	None	24–36 h
Insulin Detemir U100	3–4 h	6–8 h	24 h
Insulin Degludec U100+U200	30–90 min	None	42 h

Table adapted from Lee and Yoon (2021) Onset time = the time after injection when the blood-glucose-lowering effect is observed. Peak time = the time after injection where the maximum effect is reached. Duration of action = how long after injection the effect of the insulin lasts.

For regulatory approval of new insulins, data on efficacy as measured by the reduction of HbA1c [HbA1c is an estimate for the average blood glucose over the previous 3 months (World Health Organization, 2011)] and safety (mainly hypoglycemia) are required. This is obtained in studies designed as parallel treat-to-target trials to show non-inferiority (Pedersen-Bjergaard and Thorsteinsson, 2017). Thus, insulin doses are titrated according to specific plasma-glucose targets at specific time points. Most trials aim to demonstrate non-inferiority in HbA1c reduction. Under this circumstance, a potential benefit of a new insulin is sought in the safety data, i.e., reducing the risk of hypoglycemia. However, to reduce the risk of adverse study outcomes, participants at high risk of episodes of severe hypoglycemia are most often excluded from these trials, which has several impacts on study outcomes (Holleman et al., 1997; Hermansen et al., 2004; Heller et al., 2012). Firstly, the evidence provided does not cover the treatment of people at increased risk of severe hypoglycemia. Secondly, the rates of hypoglycemia in the trials are low, and the studies are often underpowered to show differences in the risk of severe hypoglycemia. Thirdly, the absolute differences in hypoglycemia incidence and, consequently, the pharmacoeconomic benefits of new insulins are low and may underestimate their effect in the broad type 1 diabetes population (Elliott et al., 2016).

This review will summarize the development of insulin analogs, discuss the results of insulin trials in people with MDI-treated type 1 diabetes at increased risk of hypoglycemia, and compare these results with studies in people at low risk of hypoglycemia.

## 2 Insulin treatment options

In 1922, the first purified animal-extracted insulin was successfully injected into a 14-year-old boy with diabetes (Hirsch et al., 2020; Bolli et al., 2022). In 1936, it was discovered that the addition of protamine to insulin resulted in a prolonged glucose-lowering effect (Hirsch et al., 2020; Falcetta et al., 2022). In 1946, Neutral Protamine Hagedorn (NPH) insulin was developed by adding zinc to protamine insulin, resulting in a longer duration of action (Lee and Yoon, 2021). Since then, purified animal-extracted insulin underwent continuous improvements until 1982, when recombinant DNA technology led to the synthesis of human insulin, which was approved for use in humans by the U.S. Food and Drug Administration (FDA) (Lee and Yoon, 2021).

### 2.1 Rapid-acting insulin analogs

The first insulin analog approved by the FDA was rapid-acting insulin lispro in 1996 (Falcetta et al., 2022). Compared to regular human insulin, the absorption from the injection site into the circulation is faster, leading to an onset of glucose-lowering actions after 10–15 min. The time-to-peak effect is also shorter, and the duration of the glucose-lowering activity is briefer (DiMarchi et al., 2008), resulting in a more effective lowering of postprandial hyperglycemia while also reducing the risk of post-absorptive hypoglycemia (Davey et al., 1997). In 1999, a new rapid-acting insulin analog—insulin aspart—was approved, followed by insulin glulisine in 2004. Both insulins have similar pharmacokinetic and pharmacodynamic effects as insulin lispro. Later, ultra-rapid-acting insulins were developed. In 2017, the FDA approved faster-acting insulin aspart, formulated by adding niacinamide for even faster absorption, with the onset of action occurring 5 min earlier than for regular insulin aspart (Hirsch et al., 2020; Wong and Kroon, 2021). In 2020, FDA approved ultra-rapid lispro, where prostinil and citrate are added to improve the absorption through local vasodilation and vascular permeability (Wong and Kroon, 2021). This results in the onset of action occurring after 2 min compared to the 10–15 min of regular insulin lispro.

### 2.2 Long-acting insulin analogs

In 2000, the first long-acting insulin analog, insulin glargine U100, was introduced (Lee and Yoon, 2021). It is released slowly into the circulation, leading to a glucose-lowering effect of approximately 24 h, with the onset of action after 1–2 h without a pronounced peak in effect (McKeage and Goa, 2001). This lower peak activity reduced the risk of hypoglycemia (Dunn et al., 2003). Five years later, the second long-acting insulin analog, insulin detemir U100, was released (Gallegos Aragon et al., 2019). It has an onset of action after 3–4 h with a peak effect after 6–8 h. It has a dose-dependent duration of glucose-lowering effect: approximately 20 h with doses ≥0.4 U/kg and 14 h with doses ≤0.4 U/kg, and therefore most people require twice daily dosing (Falcetta et al., 2022). Insulin degludec was released onto the market in 2015 and comes in two concentrations, U100 and U200 (Owens et al., 2019). Although the U200 insulin allows for a smaller injection volume, the pharmacokinetics and duration of action are almost similar. They both have a glycemic lowering effect of up to 42 h with onset of action after 30–90 min without a pronounced peak. A more concentrated insulin glargine (U300) was released in 2015 (Lee and Yoon, 2021). Although the chemical structure is identical to insulin glargine U100, the pharmacokinetics differ. Insulin glargine U300 has a slower release rate at the injection site, which results in a more prolonged onset of action and a duration of the glucose lowering effect of up to 36 h.

### 2.3 Other treatment options

In addition to the abovementioned human insulins and insulin analogs, several premixed insulins containing a combination of rapid-acting and intermediate-/long-acting insulins are also available (Mathieu et al., 2017; Lee and Yoon, 2021). These currently include mixtures of regular human insulin and NPH insulin, insulin lispro and insulin lispro protamin, insulin aspart and insulin aspart protamin, and insulin aspart and insulin degludec. These mixtures have the advantage and convenience of fewer injections, but due to the inability to adjust each component according to different insulin requirements during sickness, physical activity, meal composition, etc., they do not have the same flexibility as the conventional basal-bolus regimen and are consequently less used to treat type 1 diabetes (Mathieu et al., 2017).

Insulin pumps are devices for insulin delivery, mimicking physiological insulin secretion with a subcutaneous continuous pre-programmed basal release rate of rapid-acting insulin combined with user-initiated prandial boluses based on carbohydrate calculations (Kesavadev et al., 2020). Human insulin, as well as rapid-acting insulin analogs, can be used in insulin pumps, but in general, rapid-acting insulin analogs are considered the preferred choice (Pozzilli et al., 2016). With hybrid-closed-loop systems, treatment becomes even closer to physiological insulin release due to the combination of a subcutaneous continuous glucose monitoring (CGM) system with a CSII pump, which, through algorithms automatically adjusts the amount of insulin delivered (Jendle and Reznik, 2023).

## 3 Randomized controlled trial results on insulin analogs in high-risk patients

As previously mentioned, people at high risk of severe hypoglycemia are often excluded from phase III insulin trials, thus, the evidence of treatment differences in people at high risk of severe hypoglycemia is limited. So far, a total of four trials have included people at increased risk of severe hypoglycemia (Table 2).

**TABLE 2:** Overview of studies including people with type 1 diabetes at increased risk of hypoglycemia.

Study	Number of participants	Inclusion criteria	Design	Titration protocol	Run-in/cross-over period	Maintenance period	Insulins	Hypoglycemia definition	Primary endpoint
Ferguson et al. (Ferguson et al., 2001)	39 (ITT)	≥2 severe hypoglycemic episodes in the past 2 years and IAH for at least 2 years	Open-label, randomized, cross-over design	No	4 weeks	2 × 24 weeks	Insulin lispro vs. regular human insulin	BGM ≤3.5 mmol/L	Mild and severe hypoglycemia
	33 (PP)								
HypoAna (Kristensen et al., 2012b; Pedersen-Bjergaard et al., 2014a; Agesen et al., 2016; Pedersen-Bjergaard et al., 2016; Agesen et al., 2018)	141 (ITT)	≥2 severe hypoglycemic episodes in the past year	Multicenter PROBE^a^ cross-over	No	3 months	2 × 9 months	Insulin detemir + Insulin aspart vs. NPH insulin + regular human insulin	BGM ≤3.9 mmol/L	Severe hypoglycemia
	114 (PP)								
SWITCH-1 (Lane et al., 2017; Evans et al., 2018)	501 (ITT)	1 or more of the following:	Randomized, double-blinded, cross-over, multicenter, treat-to-target clinical trial	Yes	16 weeks	2 × 16 weeks	Insulin degludec vs. insulin glargine U100	BGM <3.1 mmol/L	Total severe or BGM confirmed symptomatic hypoglycemia
	395 (PP)	• ≥1 severe hypoglycemic episode in the past year							
		• eGFR 30–59 mL/min/1.73m^2^							
		• Hypoglycemia unawareness							
		• Diabetes duration >15 years							
		• Hypoglycemic episode in the past 12 weeks							
HypoDeg (Agesen et al., 2019; Brøsen et al., 2022; Pedersen-Bjergaard et al., 2022; Brøsen et al., 2023)	149 (ITT)	≥1 severe nocturnal hypoglycemic episodes in the past 2 years	Multicenter PROBE^a^ cross-over	No	3 months	2 × 9 months	Insulin degludec vs. insulin glargine U100	Level 1: BGM ≤ 3.9 mmol/L	Nocturnal symptomatic hypoglycemia
	132 (PP)							Level 2: BGM ≤ 3.0 mmol/L	

ITT, intention-to-treat population, PP, per protocol population; BGM, blood glucose monitoring; IAH, impaired awareness of hypoglycemia.

^a^ PROBE, prospective, randomized, open, blinded endpoint design.

### 3.1 Insulin lispro versus regular human insulin

Treatment with fast-acting insulin lispro was compared to treatment with regular human insulin during a 1-year open-label, randomized cross-over study by Ferguson et al. from 2001 (Ferguson et al., 2001). The study consisted of a four-week run-in period in which all participants were treated with regular human insulin in combination with NPH insulin. The run-in period was followed by two 24-week maintenance treatment periods of either insulin lispro and NPH insulin or regular human insulin and NPH insulin. Participants were offered assistance with dose adjustment at the beginning of each treatment phase but were allowed to adjust their insulin doses on a daily basis. The participants were not requested or recommended any formal blood glucose targets. The participants included in the study had experienced at least two episodes of severe hypoglycemia in the preceding 2 years and had had impaired awareness of hypoglycemia for at least 2 years. A total of 33 participants completed the study. Hypoglycemia was defined as blood glucose values ≤ 3.5 mmol/L and all symptomatic events were included whether or not blood glucose was measured. Lastly, all events of severe hypoglycemia, defined by requiring third-party assistance to restore blood glucose, were included. The primary endpoint was the incidence of severe hypoglycemia. No statistically significant difference in severe hypoglycemia was found between treatments, but there seemed to be a tendency towards a lower incidence of severe hypoglycemia in the insulin lispro treatment group (*p* = 0.087) (Table 3). Fewer severe nocturnal hypoglycemic episodes were observed during treatment with insulin lispro (Table 3). This was found both during the early (00 h–04 h) and later (04 h–08 h) parts of the night, but a statistically significant reduction was not found in either nocturnal time period (*p* = 0.11). For non-severe hypoglycemic episodes no statistically significant difference was found between treatment with insulin lispro and regular human insulin. Glycemic control as determined by HbA1c was similar between the two treatments.

**TABLE 3:** Comparison of insulin lispro vs. human insulin trials.

	Ferguson et al. (2001)		Holleman et al. (1997)	
	RRR (%) with insulin lispro	ARR (E/PY) with insulin lispro	RRR (%) with insulin lispro	ARR (E/PY) with insulin lispro
All-day (Davey et al., 1997) hypoglycemia				
Total hypoglycemia	3.7	−2.7	4.1	2.19
Non-severe hypoglycemia	6.8	−4.6	3.2	1.68
Severe hypoglycemia	34.5	1.91	37.9*	0.51
Nocturnal hypoglycemia				
Total hypoglycemia	-	-	43.6**	3.13
Severe hypoglycemia	46.8	1.45	-	-

Abbreviations: RRR, relative rate reduction; ARR, absolute rate reduction, E/PY, events per person-year.

* = p< 0.05, ** = p< 0.001.

Nocturnal = 00 h–08 h (Ferguson), 00 h–06 h (Holleman).

Note: RRR and ARR are estimated from the number of events, follow-up time, and number of participants. Results from Ferguson et al. are from PP population. Results from Holleman et al. are from ITT population.

Though Ferguson et al. found no statistically significant difference in severe hypoglycemia between the two treatments, there was a trend towards a lower rate of severe hypoglycemia during treatment with insulin lispro, mainly due to fewer nocturnal events (Ferguson et al., 2001). Holleman et al. explored treatment differences in people with type 1 diabetes without increased risk of hypoglycemia during treatment with insulin lispro compared to regular human insulin. This study demonstrated a significant reduction in total episodes of nocturnal hypoglycemia and all-day severe hypoglycemia during treatment with insulin lispro (Holleman et al., 1997). From the number of events, follow-up time, and number of participants, we have estimated relative and absolute rate reduction in the two trials (Table 3). Although the results from Ferguson et al. did not reach statistical significance due to a low number of participants, the RRR for severe hypoglycemia is comparable to the study by Holleman et al. (34.5% vs. 37.9%), but with a larger ARR in the study by Ferguson et al., which included people at increased risk of hypoglycemia (1.91 vs. 0.51 E/PY).

### 3.2 Insulin detemir + insulin aspart versus NPH insulin + human regular insulin

The HypoAna Trial—a multicenter, prospective, randomized, open, blinded-endpoint cross-over study—from 2012 examined the difference in the occurrence of severe hypoglycemia during treatment with insulin detemir and insulin aspart (analog insulins) compared to NPH insulin and human regular insulin (human insulins) (Kristensen et al., 2012b). The study lasted 2 years, consisting of two one-year treatment periods with a three-month run-in/cross-over period followed by a nine-month maintenance period. Apart from the main trial there were two substudies: One overnight substudy where participants had their plasma glucose measured every hour during the night (Kristensen et al., 2017), and a substudy where participants were equipped with a blinded CGM for 3 days (Agesen et al., 2018). Both substudies were performed after 6 and 12 months in each treatment arm. The participants were people with type 1 diabetes who had experienced two or more episodes of severe hypoglycemia during the last year, and 114 subjects completed the trial. Hypoglycemia was defined as a blood glucose value ≤ 3.9 mmol/L. The primary endpoint was episodes of severe hypoglycemia. The glycemic target was the maintenance of baseline HbA1c to avoid an excessive risk of hypoglycemia, which was overall maintained during the entire study period, but a small, yet statistically significant, reduction of 1.4 mmol/mol during treatment with insulin analogs in the maintenance periods (*p* < 0.05) (Pedersen-Bjergaard et al., 2014a).

The number of reported all-day severe hypoglycemic episodes was lower during treatment with insulin analogs than treatment with human insulin. This was demonstrated by an absolute rate reduction (ARR) of 0.5 episodes per patient-year (*p* = 0.01) during treatment with insulin analogs (Pedersen-Bjergaard et al., 2014a). The reduction of severe hypoglycemia during treatment with insulin analogs seemed to be largest during the night (from 23 h to 7 h). Treatment with insulin analogs also led to a relative rate reduction (RRR) of 6% for all-day non-severe events with an ARR of 4.6 events per patient-year (E/PY) (*p* < 0.005) (Agesen et al., 2016). However, an increased rate of daytime asymptomatic hypoglycemia was found in the analog treatment arm. For nocturnal non-severe hypoglycemia, the RRR was 39% for treatment with insulin analogs corresponding to an ARR of 4.2 events per patient-year (*p* < 0.0001), and when divided into asymptomatic and symptomatic nocturnal hypoglycemia, the RRR was 28% (*p* < 0.001) and 48% (*p* < 0.0001), respectively (Agesen et al., 2016). The reduced rate of hypoglycemia was consistent throughout the night. These results from the main trial were supported by results from the overnight substudy in which a higher overall incidence of nocturnal hypoglycemia, measured by hourly plasma glucose, was found in the human insulin treatment arm (Kristensen et al., 2017). Results from the CGM-substudy supported the results of higher rates of hypoglycemic events during treatment with human insulin (Agesen et al., 2018). However, when the CGM data were compared to blood glucose monitor (BGM) levels, the ARR for all-day non-severe hypoglycemic was more than ten times higher in the CGM data (4.6 vs. 52 E/PY) (Table 6).

A study by Hermansen et al. compared treatment with insulin analogs (insulin detemir/insulin aspart) to human insulins (NPH insulin/regular human insulin) in people with type 1 diabetes without increased risk of hypoglycemia (Hermansen et al., 2004). They reported a 21% lower rate of hypoglycemia during treatment with insulin analogs (*p* < 0.05) (Table 4). The rate for nocturnal non-severe hypoglycemia was 54% lower during treatment with insulin analogs than human insulins, equivalent to an ARR of 3.4 events per patient-year (*p* < 0.001). These results correspond to the data from the HypoAna trial, where the RRR for nocturnal non-severe hypoglycemia was 39% during treatment with insulin analogs, translating into an ARR of 4.2 E/PY (Agesen et al., 2016). While the ARR for nocturnal non-severe hypoglycemia was comparable between the two studies, the results for severe hypoglycemia differed. The HypoAna trial reported an ARR of 0.51 events per person-year for treatment with insulin analogs compared to human insulins, whereas Hermansen et al. found no statistically significant difference in the rate of severe hypoglycemia between the two treatment groups.

**TABLE 4:** Comparison of insulin detemir + insulin aspart vs. NPH insulin + regular human insulin trials.

	HypoAna (Pedersen-Bjergaard et al., 2014a; Agesen et al., 2016)		Hermansen et al. (Hermansen et al., 2004)	
	RRR (%) with insulin analogs	ARR (E/PY) with insulin analogs	RRR (%) with insulin analogs	ARR (E/PY) with insulin analogs
All-day (24 h) hypoglycemia				
Total hypoglycemia	-	-	21*	10.3
Non-severe hypoglycemia	6*	4.6	21*	7.4
Symptomatic hypoglycemia	4	0.6	-	-
Asymptomatic hypoglycemia	−4	0.3	-	-
Severe hypoglycemia	29*	0.51	11	0.08
Daytime hypoglycemia				
Non-severe hypoglycemia	1	1	-	-
Symptomatic hypoglycemia	2	0	-	-
Asymptomatic hypoglycemia	−13*	−1	-	-
Nocturnal hypoglycemia				
Total nocturnal hypoglycemia	-	-	55**	4.9
Non-severe hypoglycemia	39**	4.2	54**	3.4
Symptomatic hypoglycemia	48**	1.6	-	-
Asymptomatic hypoglycemia	28**	1.1	-	-
Severe hypoglycemia	-	-	83*	0.3

Abbreviations: RRR, relative rate reduction; ARR, absolute rate reduction, E/PY, events per person-year.

* = p< 0.05, ** = p< 0.001.

Nocturnal: 23 h–06 h (Hermansen), 23 h–07 h (HypoAna).

Note: ARR was not given in the Hermansen article and was estimated from the number of events, follow-up time, and number of participants. ARR was not provided for statistically insignificant results in the HypoAna articles and is calculated from the given E/year for each treatment. Results for HypoAna non-severe hypoglycemia are from PP population. Results for HypoAna severe and from Hermansen et al. are from ITT population.

### 3.3 Insulin degludec versus insulin glargine U100

The SWITCH-1 randomized clinical trial was a double-blinded, two-period cross-over, multicenter, treat-to-target trial that examined treatment with insulin degludec and insulin glargine U100 in 501 participants (Lane et al., 2017). The study lasted 65 weeks, consisting of two 32-week treatment periods followed by 1 week of follow-up. The study was a treat-to-target trial using a set protocol for insulin titration for prandial as well as basal insulin. People with type 1 diabetes with at least one risk factor for developing hypoglycemia were included. The target was a fasting plasma glucose of 3.9–5 mmol/L and a preprandial blood glucose of 3.9–6 mmol/L. The primary endpoint was the total rate of severe or blood-glucose-confirmed symptomatic hypoglycemia. Hypoglycemia was defined as symptomatic events with blood glucose <3.1 mmol/L and severe hypoglycemic events at which the subject required third-party assistance to restore glucose levels.

During treatment with insulin degludec, the rate of severe or blood-glucose-confirmed symptomatic hypoglycemic events was significantly lower compared to insulin glargine U100 with an RRR of 11%, translating to an ARR of 1.3 events per patient-year (*p* < 0.001) (Lane et al., 2017). Nocturnal severe or blood glucose-confirmed symptomatic hypoglycemic events were fewer in the insulin degludec treatment arm, with an RRR of 36%, translating to an ARR of 0.62 per patient-year (*p* < 0.001). The rate of severe hypoglycemia was also lower in the insulin degludec group compared to the insulin glargine U100 group with an RRR of 35%, corresponding to an ARR of 0.14 severe hypoglycemic events per patient-year (*p* < 0.01). HbA1c was similar between the treatments, but fasting BGM values decreased significantly with insulin degludec treatment with an assessed treatment difference of 0.94 mmol/L (Lane et al., 2017). A *post hoc* analysis found that the incidence of hypoglycemia was positively associated with a reduction in HbA1c in both treatment groups. However, the analysis also showed that a given reduction in HbA1c was associated with a smaller relative increase in the incidence of hypoglycemia during treatment with insulin degludec compared to insulin glargine U100 (Philis-Tsimikas et al., 2020).

The HypoDeg study—a multicenter, prospective, randomized, open, blinded-endpoint cross-over study—also examined the difference in treatment with insulin degludec compared to insulin glargine U100 (Agesen et al., 2019). Insulin aspart was used as bolus treatment during both treatment periods. The study lasted 2 years, each year consisting of a three-month run-in/cross-over and a nine-month maintenance period. Apart from the main trial two substudies were performed. One substudy was an overnight substudy where participants had their plasma glucose measured every hour during the night (Brøsen et al., 2023). In the other substudy the participants were equipped with a blinded CGM for 6 days. Both substudies were performed after 6 and 12 months in each treatment arm (Brøsen et al., 2022). The study included 149 people with type 1 diabetes with at least one episode of severe nocturnal hypoglycemia in the preceding 2 years. The glycemic target of the HypoDeg trial was to maintain baseline HbA1c during both treatment periods, which was obtained, as no significant difference in HbA1c was found between the two treatments (Pedersen-Bjergaard et al., 2022). The primary endpoint was the number of nocturnal symptomatic hypoglycemic episodes, and the secondary endpoints were incidences of severe hypoglycemia (total, daytime, nocturnal). Hypoglycemia was classified into two levels: a BGM ≤ 3.9 mmol/L (level 1) or ≤ 3.0 mmol/L (level 2).

A lower rate of nocturnal symptomatic hypoglycemia was found during treatment with insulin degludec compared to insulin glargine U100, with an RRR of 28% for level 1 hypoglycemia (*p* < 0.05) and 37% for level 2 hypoglycemia (*p* < 0.005) (Pedersen-Bjergaard et al., 2022). This corresponds to an ARR of 1.04 and 0.84 episodes per person-year, respectively. For severe hypoglycemia the RRR was 35% for treatment with insulin degludec compared to insulin glargine, translating into an ARR of 0.3 episodes per person-year (*p* < 0.05). This difference was primarily due to a lower incidence of nocturnal severe hypoglycemic episodes, whereas the incidence of daytime severe hypoglycemia was similar between the two treatment groups. The incidence of all-day symptomatic hypoglycemia was similar between the two treatment groups (Pedersen-Bjergaard et al., 2022). During daytime, however, the rate of symptomatic level 1 hypoglycemia was increased by 28% in the insulin degludec treatment group compared to insulin glargine U100 (*p* = 0.01). No difference in level 2 hypoglycemia was found between the two treatments. Results from the CGM-substudy supported the results from the main trial, but the relative rate reduction for hypoglycemia during treatment with insulin degludec was even higher (Brøsen et al., 2022). A 36% RRR was found for level 1 nocturnal hypoglycemia (*p* < 0.01), and for level 2 nocturnal hypoglycemia there was a RRR of 53% (*p* < 0.001). The corresponding ARRs were 44 and 39 events per patient-year, respectively. The differences in ARR from the findings of the main trial were mainly due to a more extensive detection of nocturnal asymptomatic hypoglycemic events during CGM. These findings were supported by results from the overnight substudy (Brøsen et al., 2023). Based on these profiles, treatment with insulin degludec resulted in a lower risk of level 1 and level 2 nocturnal hypoglycemia compared to insulin glargine U100. The BEGIN trial was a phase III trial comparing insulin degludec and insulin glargine U100 in people with type 1 diabetes without increased risk of hypoglycemia (Heller et al., 2012). No significant difference in all-day hypoglycemia between treatments was found, which corresponds to the findings of the HypoDeg trial (Heller et al., 2012; Pedersen-Bjergaard et al., 2022) but differs from the SWITCH-1 trial, where an 11% lower risk of all-day hypoglycemia was found during treatment with insulin degludec (Table 5) (Lane et al., 2017). Nocturnal BGM-confirmed hypoglycemia was reduced in all three trials with a comparable RRR of 27%–37%, but the ARR was highest in the HypoDeg trial with an ARR of 0.84 events per patient-year (Heller et al., 2012; Lane et al., 2017; Pedersen-Bjergaard et al., 2022). The rate of severe hypoglycemia was low in the BEGIN trial, and no difference between the treatments was found, which was possibly due to the exclusion of people at increased risk of severe hypoglycemia. In contrast, the HypoDeg and the SWITCH-1 trials, both including participants at increased risk of severe hypoglycemia, found significant reductions in all-day severe hypoglycemia with a risk reduction of about 35%, but the ARR was almost twice as high in the HypoDeg trial than in the SWITCH-1 trial (0.3 vs. 0.14 E/PY).

**TABLE 5:** Comparison of insulin degludec vs. insulin glargine U100 trials.

	SWITCH-1 (Lane et al., 2017)		HypoDeg (Pedersen-Bjergaard et al., 2022)		BEGIN (Heller et al., 2012)	
	RRR (%) with insulin degludec	ARR (E/PY) with insulin degludec	RRR (%) with insulin degludec	ARR (E/PY) with insulin degludec	RRR (%) with insulin degludec	ARR (E/PY) with insulin degludec
All-day (24 h) hypoglycemia						
Total hypoglycemia	11**	1.3	-	-	−2	-
Non-severe symptomatic hypoglycemia (level 1)	-	-	−15	−3.1	-	-
Non-severe symptomatic hypoglycemia (level 2)	-	-	2	1.1	-	-
Severe hypoglycemia	35*	0.14	35*	0.3	-	-
Daytime (06h–00 h) hypoglycemia						
Non-severe symptomatic hypoglycemia (level 1)	-	-	−28*	−4.8	-	-
Non-severe symptomatic hypoglycemia (level 2)	-	-	−10	−0.3	-	-
Severe hypoglycemia	-	-	10	0.07	-	-
Nocturnal (00h–06 h) hypoglycemia						
Total nocturnal hypoglycemia	36**	0.62	-	-	27*	-
Non-severe symptomatic hypoglycemia (level 1)	-	-	28*	1.04	-	-
Non-severe symptomatic hypoglycemia (level 2)	-	-	37*	0.84	-	-
Severe hypoglycemia	-	-	52	0.19	-	-

Abbreviations: RRR, relative rate reduction; ARR, absolute rate reduction, E/PY, events per person-year.

* = p< 0.05, ** = p< 0.001.

Level 1 hypoglycemia: ≤3.9 mmol/L. Level 2 hypoglycemia: ≤3.1 mmol/L (SWITCH-1, BEGIN) ≤3.0 mmol/L (HypoDeg).

Note: ARR was not given in the BEGIN trial, and no data was available to make an estimate hereof. Results from all three studies are from ITT population.

## 4 Discussion

Only a few studies on treatment with insulin analogs in type 1 diabetes have included people at high risk of severe hypoglycemia, though it may potentially be those people benefiting the most from insulin analogs. These studies generally confirm the results of phase III trials and trials excluding people at high risk of hypoglycemia (Holleman et al., 1997; Hermansen et al., 2004; Heller et al., 2012) in terms of similar HbA1c reduction or maintenance and relative treatment differences in risk of hypoglycemia but with more considerable absolute differences in hypoglycemia risk reduction. In general, the endpoints that were used for inclusion in the trials, e.g., severe hypoglycemia in the lispro study (Ferguson et al., 2001) and nocturnal severe hypoglycemia in the HypoDeg trial (Pedersen-Bjergaard et al., 2022)—showed the largest differences in ARR when compared to the results from the phase III trials.

These findings have important clinical and regulatory implications for treatment with insulin. Firstly, the absolute treatment effects observed in phase III trials grossly underestimate the clinical effects, especially for severe and nocturnal hypoglycemia, as well as for asymptomatic hypoglycemia, that can be expected when implementing the treatment in the broader type 1 diabetes population (Elliott et al., 2016).

Secondly, the pharmacoeconomic effect of implementing new insulins is underestimated when people at high risk of hypoglycemia are excluded from clinical trials. This is primarily due to the pharmacoeconomic effect being closely related to the reduction of exposure to hypoglycemia, particularly severe hypoglycemic events. Both the HypoAna and SWITCH-1 trials demonstrated this. The HypoAna trial showed that although the total cost was larger for treatment with insulin analogs, the analog regimen was considered more cost-effective than the human insulin regimen during 1 year of treatment due to lower rates of severe and nocturnal hypoglycemia (Pedersen-Bjergaard et al., 2016). The SWITCH-1 trial also reported that the cost of treatment with insulin degludec was higher compared to insulin glargine U100, however, treatment with insulin degludec was more cost-effective as a result of the reduction in the rates of nocturnal and severe hypoglycemia (Evans et al., 2018).

In principle, the resulting knowledge gap on treatment differences in people at increased risk of severe hypoglycemia can be covered by real-world data obtained during implementation in clinical practice. However, because non-severe events can be asymptomatic and may go unnoticed by the participant—especially among people with impaired awareness of hypoglycemia—the frequency can be underestimated in real-world data (Elliott et al., 2016). Moreover, some people may not consider non-severe events significant enough to report to their physician and may, therefore, go unreported in real-world data (Östenson et al., 2014). The same is true for nocturnal events, which may go unnoticed by the person with diabetes and their next of kin, thus being underreported. The rate of severe hypoglycemia reported to physicians may also not be entirely representative as some people may deliberately withhold the information due to the fear of not being able to obtain and maintain their driver’s license (Pedersen-Bjergaard et al., 2014b). Using hospital records as an indication of the number of severe hypoglycemic events is not representative since only a fraction of severe events are treated in the hospital (Pedersen-Bjergaard and Thorsteinsson, 2017). Another point to consider when using real-world data is that hypoglycemia is a leading indicator to switch to other insulins and will, as such, impose a major bias by indication (Kristensen et al., 2012a). Therefore, dedicated randomized clinical trials, such as Phase IV effectiveness trials, which include people with a high risk of hypoglycemia are warranted.

However, studies comparing the effects of different insulin regimens in people at increased risk of hypoglycemia must be carefully designed (Pedersen-Bjergaard and Thorsteinsson, 2017). People with type 1 diabetes at increased risk of hypoglycemia are often excluded from clinical trials to reduce the risk of single subjects distorting the results because of the skewed distribution of hypoglycemia (Pedersen-Bjergaard et al., 2004; Kristensen et al., 2012a; Amiel, 2021). By using a cross-over design, all participants contribute with data to both treatments, minimizing the impact of between-person variability and limiting the effect of the skewed hypoglycemia distribution. The importance of this was demonstrated in a trial comparing treatment with faster-acting aspart compared to regular insulin aspart in insulin pump users (Klonoff et al., 2019). In this parallel-group trial allowing participation of people with increased risk of severe hypoglycemia, an imbalance in the number of severe hypoglycemia between the two treatments was likely due to a disproportionate distribution of participants with increased rates of severe hypoglycemia. Thus, randomization of three participants with recurrent severe hypoglycemia to the faster aspart treatment group resulted in a skewed distribution of severe hypoglycemia, with the three participants accounting for almost half of the total number of severe episodes.

Another important learning point from cross-over studies is that individualization of insulin therapy may improve glycemic control and reduce hypoglycemia. A *post hoc* analysis of the HypoAna trial examining the incidence of severe hypoglycemia and HbA1c levels for each participant demonstrated this (Pedersen-Bjergaard et al., 2017). When combining these two outcomes, it was found that 49% of the subjects had a superior outcome with insulin analogs, 25% had superior outcomes with human insulins, and 26% had similar outcomes with the two treatment regimens, meaning that the overall trial outcome did not apply to all participants. When the best possible treatment was selected for each subject, the rate of severe hypoglycemia was reduced to 0.7 episodes per patient-year, as opposed to 1.1 for the insulin analog regimen and 1.6 for treatment with human insulins.

When designing clinical trials comparing insulins, insulin titration algorithms must be considered. In the trial by Ferguson and the HypoAna and HypoDeg trials, insulin doses were adjusted individually according to baseline HbA1c to avoid imposing further hypoglycemia risk on the participants by applying a titration regimen while also resembling everyday clinical practice more (Kristensen et al., 2012b; Agesen et al., 2019). In contrast, the SWITCH-1 trial used a set protocol for insulin titrating with strict fasting glucose targets, raising concerns for exceeding common clinical practice and, as a result, boosting the number of endpoints throughout the study period (Seaquist and Chow, 2017; Zulewski and Keller, 2017; Tentolouris et al., 2018).

Though carefully considering the design of the clinical trial can help limit biases and avoid the impact of the skewed distribution of hypoglycemia among participants, there are still some potential factors that may impact the results of the trials. First of all, it is important to consider the potential attention bias, as the participants of a clinical trial may become more aware of their patterns of hypoglycemia and therefore adjust their behavior (e.g., meals, physical activity, glycaemic level) to avoid hypoglycemic events. Another factor that may impact study results is regression towards the mean which can lead to a lower incidence of hypoglycemia during the study periods than what was expected as indicated by the baseline incidence. Lastly, there is a potential for differential dropouts between treatment groups, and therefore it is important to perform analyses on both the per-protocol population and the intention-to-treat population.

The Kobe Best Basal insulin study has examined treatment differences between insulin degludec and insulin glargine U300 in people with type 1 diabetes without increased risk of severe hypoglycemia (Miura et al., 2018). No significant difference in blood glucose levels (BGM) or frequency of hypoglycemia was found between the two treatments, and no severe hypoglycemic events were reported during the study period (Miura et al., 2020). However, treatment differences between insulin degludec and insulin glargine U300 remain tentative in people with type 1 diabetes who are at increased risk of severe hypoglycemia and should be explored in future studies.

The InRange trial also explored differences in treatment with insulin degludec U100 and insulin glargine U300 in people with type 1 diabetes using CGM data (Battelino et al., 2020). Patient-reported hypoglycemia and hypoglycemic episodes recorded by CGM were comparable between the two treatment groups for all time periods and both severe and non-severe episodes (Battelino et al., 2023). However, CGM data revealed a 2–6 times higher rate of hypoglycemic events than the patient-reported events (Battelino et al., 2023). The HypoAna and the HypoDeg trials had CGM substudies, confirming the findings from the main trials, but due to the higher detection of asymptomatic hypoglycemia—especially during the night—the treatment differences were even greater when using CGM data (Agesen et al., 2018; Brøsen et al., 2022) (Tables 6, 7). This emphasizes the importance of using CGM-derived endpoints in intervention trials in order to assess the actual rate of asymptomatic hypoglycemia, especially when including people at increased risk of hypoglycemia.

**TABLE 6:** Comparison of BGM and CGM results—HypoAna trial.

	BGM (Agesen et al., 2016)		CGM (Agesen et al., 2018)	
	RRR (%) with insulin analogs	ARR (E/PY) with insulin analogs	RRR (%) with insulin analogs	ARR (E/PY) with insulin analogs
All-day hypoglycemia				
Total hypoglycemia	6*	4.6	20	52
Symptomatic hypoglycemia	4	0.6	−10	−5.2
Asymptomatic hypoglycemia	−4	0.3	30	52
Daytime hypoglycemia				
Total hypoglycemia	1	1	0	5.2
Symptomatic hypoglycemia	2	0	−40	−5.2
Asymptomatic hypoglycemia	−13*	−1.0	20	15.6
Nocturnal hypoglycemia				
Total hypoglycemia	39**	4.2	40*	46.8
Symptomatic hypoglycemia	48**	1.6	80	5.2
Asymptomatic hypoglycemia	28**	1.1	40*	36.4

Abbreviations: BGM, blood glucose monitoring; CGM, continuous glucose monitoring; RRR, relative rate reduction; ARR, absolute rate reduction, E/PY, events per person-year.

* = p< 0.05, ** = p< 0.001.

**TABLE 7:** Comparison of BGM and CGM results—HypoDeg trial.

	BGM (Pedersen-Bjergaard et al., 2022)		CGM (Brøsen et al., 2022)	
	RRR (%) with insulin degludec	ARR (E/PY) with insulin degludec	RRR (%) with insulin degludec	ARR (E/PY) with insulin degludec
All day hypoglycemia				
Total hypoglycemia (level 1)	-	-	12	43.7
Symptomatic hypoglycemia	−15	−3.1	11	8.8
Asymptomatic hypoglycemia	-	-	9	29.6
Total hypoglycemia (level 2)	-	-	34**	55.1
Symptomatic hypoglycemia	2	1.1	48*	9.4
Asymptomatic hypoglycemia	-	-	25*	25.5
Daytime (07h–23 h) hypoglycemia				
Total hypoglycemia (level 1)	-	-	2	13
Symptomatic hypoglycemia	−23*	−4.2	−8	−5.7
Asymptomatic hypoglycemia	-	-	0	7.3
Total hypoglycemia (level 2)	-	-	24*	25.5
Symptomatic hypoglycemia	−6	0.2	49*	7.28
Asymptomatic hypoglycemia	-	-	11	2.3
Nocturnal (23h–07 h) hypoglycemia				
Total hypoglycemia (level 1)	-	-	36*	44.2
Symptomatic hypoglycemia	28*	1.75	29	8.8
Asymptomatic hypoglycemia	-	-	32*	28.6
Total hypoglycemia (level 2)	-	-	53**	39
Symptomatic hypoglycemia	34**	1.3	46	7.3
Asymptomatic hypoglycemia			52**	22.3

Abbreviations: BGM, blood glucose monitoring; CGM, continuous glucose monitoring; RRR, relative rate reduction; ARR, absolute rate reduction, E/PY, events per person-year.

* = p< 0.05, ** = p< 0.001.

Level 1 hypoglycemia: ≤3.9 mmol/L. Level 2 hypoglycemia: ≤3.0 mmol/L.

Note: ARR from the HypoDeg CGM-substudy was given as events per patient-week. To compare to BGM results, this has been calculated to events per patient-year by multiplying with 52 weeks.

In conclusion, the relative differences in the treatment effect of insulin analogs in people with type 1 diabetes at increased risk of hypoglycemia are comparable to those observed in low risk populations in phase III trial. However, the differences in the absolute treatment effect of insulin analogs are much more clinically and pharmacoeconomically significant in people with type 1 diabetes than apparent from phase III trials. As real-world data may be biased and lack high-quality data on hypoglycemia, more trials that include people at increased risk of hypoglycemia are needed.
